# A Quantitative Method to Analyze Drosophila Pupal Eye Patterning

**DOI:** 10.1371/journal.pone.0007008

**Published:** 2009-09-15

**Authors:** Ruth I. Johnson, Ross L. Cagan

**Affiliations:** Department of Developmental and Regenerative Biology, Mount Sinai Medical School, New York, New York, United States of America; University of Washington, United States of America

## Abstract

**Background:**

The Drosophila pupal eye has become a popular paradigm for understanding morphogenesis and tissue patterning. Correct rearrangement of cells between ommatidia is required to organize the ommatidial array across the eye field. This requires cell movement, cell death, changes to cell-cell adhesion, signaling and fate specification.

**Methodology:**

We describe a method to quantitatively assess mis-patterning of the Drosophila pupal eye and objectively calculate a ‘mis-patterning score’ characteristic of a specific genotype. This entails step-by-step scoring of specific traits observed in pupal eyes dissected 40–42 hours after puparium formation and subsequent statistical analysis of this data.

**Significance:**

This method provides an unbiased quantitative score of mis-patterning severity that can be used to compare the impact of different genetic mutations on tissue patterning.

## Introduction

The Drosophila compound eye has emerged as a superb tissue in which to study a variety of processes. In particular, pupal eye tissue provides opportunities to examine cell death, signaling, fate specification, cell movement, adhesion and regulation of the cytoskeleton [1]. Errors in these processes produce irregular numbers and organization of cells. These changes can consequently disrupt the precise hexagonal outlines of ommatidia and if sufficiently severe lead to rough adult eye phenotypes. As our understanding of these processes and the group of genes we study increases in sophistication, it becomes increasingly important to account for multiple components of a mutant phenotype rather than a single aspect (such as cell number). We have therefore developed a simple system to systematically analyze and record multiple components of pupal eye phenotypes. This quantitative assessment enables efficient, thorough comparison of genotypes as well as meaningful statistical analyses because each genotype is objectively ranked according to the scope and severity of mis-patterning. An earlier version of this method was successfully used to assess and validate genetic interactions between *cindr* (which encodes an adaptor protein with roles including actin regulation and endocytosis) and loci encoding actin regulators and junction components [2].

**Figure 1:**
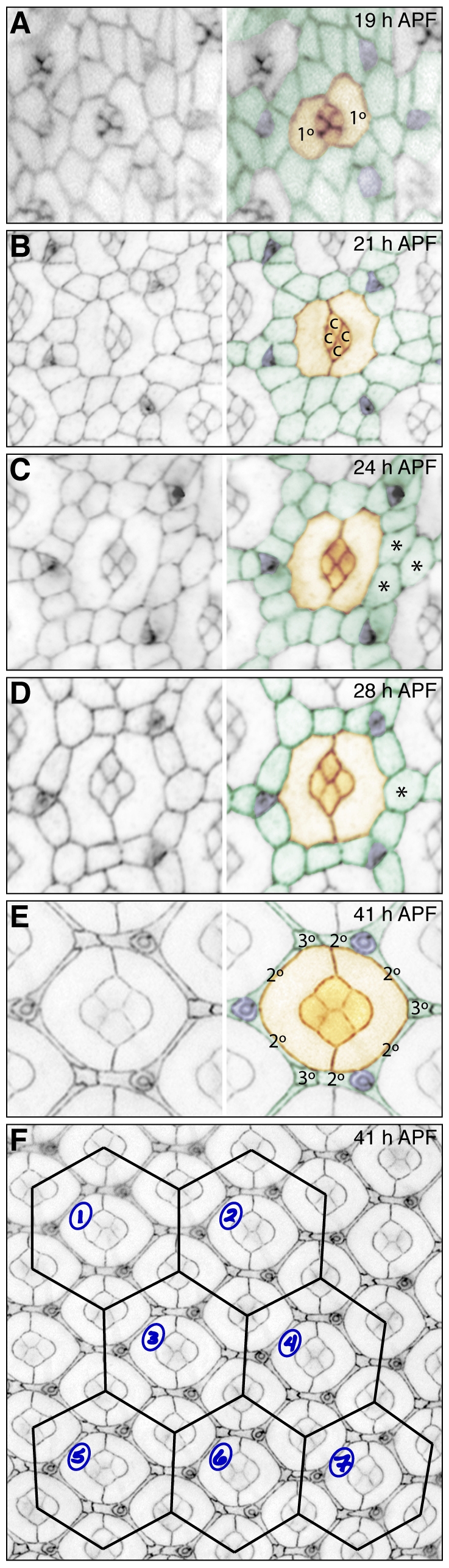
Patterning the wild type fly pupal eye. A-E. Commencing 19 h APF, two 1°s (labeled, pseudo-colored orange) encircle the central four cone cells of each ommatidium (labeled c in B, also orange). Three bristle groups (purple) position at three vertices of the final hexagon. Lattice cells (in green) gradually reorganize coming into single file at around 24 h APF. Frequently three cells occupy the 3° cell niche (asterisks in C) before this is resolved to a single cell (asterisks in D, labeled 3° in E). Excess cells are removed by apoptosis, leaving single 2° cells extending along each side of the hexagon (E). F. For analysis a hexagonal lattice is superimposed onto an image to create data points (seven shown).

The wild type fly pupal eye has a limited number of cell types [3]: eight photoreceptors that are recruited a full day earlier during the third larval instar and subsequently organized into characteristic positions within each ommatidium, bristle cell organules (composed of four different cells) and four glial-like accessory cell types that take on distinctly recognizable shapes and positions. These are the cone cells (that lie mainly above the photoreceptors with basal processes during development), the primary (1°) pigment cells (which surround the cone cells), and secondary (2°) and tertiary (3°) pigment cells that form a honeycomb lattice across the eye field enclosing and separating neighboring ommatidia. Patterning of these lattice cells occurs between 18–28 hours after puparium formation (h APF) at 25°C: a process of active cell rearrangement and programmed cell death (PCD) reorganizes these cells into their final pattern [4], [5] (Figure 1A-D). The final surface pattern is most usefully scored at 40–42 h APF (Figure 1E). Here we describe typical mutant phenotypes and a simple method to score them to comprehensively quantify mis-patterning. Photoreceptor cells are not present at the surface of the pupal retina and are not included in this analysis.

## Results

A hexagonal grid was superimposed on images of the apical profile of pupal eyes dissected at 41 h APF (Figure 1F) as follows: each hexagon was drawn to connect the centre of 6 ommatidia surrounding a central ommatidium; this field was then utilized as a single data point as we scored defects observed within each hexagonal area. One ‘point’ was awarded per defect and recorded in a spreadsheet (Microsoft Excel) and then summed to give a total number of defects per field. We found that analyzing 75 ommatidia of a genotype and then determining the average number of errors per ommatidium provided a reliable ‘ommatidial mis-patterning score’ (OMS) characteristic of that genotype; variance and standard deviation were also included. Some mutant eyes displayed position-specific defects. For example we consistently found more severe phenotypes in the posterior hemisphere of the eye when expressing an RNAi transgene. In addition bristle groupings were often observed to be missing or mis-positioned toward the periphery of the eye field. To prevent such position-dependent effects from skewing the final OMS, we routinely imaged and analyzed only the central region of the pupal eye. In addition the 75 data points were collected from images obtained from between 8 and 12 pupal eyes, and dissections were repeated to ensure that observations fairly represent each genotype. Inevitably it is also important to culture and dissect control and experimental genotypes simultaneously to allow proper comparison of age-matched phenotypes.

The following features were scored (summarized in Table 1; all phenotypes refer to the apical surface, refer to Materials and Methods for genotypes):

**Table 1:** Patterning errors scored.

Cell type	Feature scored	Score allocated	
Cone cells	Number	+1	per additional or missing cone cell
	Cell arrangement	+1	if arrangement not according to free energy minimization
	Junction orientation	+1	per incorrect orientation of cone-cell adhesion
	Cluster orientation	+1	if incorrect
1°s	Number	+1	per additional or missing cell
	Size	+1	if one 1° is ≥25% larger/smaller than the other(s)
	Junction	+1	if junctions incomplete in any way
	Cone contact cells	+1	for each 2° or 3° cell that touches the central cone cells due to disrupted 1°∶1° cell junction(s)
Cone & 1°s	Rotation	+1	if the central ommatidial grouping is mis-rotated by ≥30°
Bristle group	Missing or mis-positioned	+1	per missing or mis-positioned bristle cell group (max of 3 points can be allocated per data point)
	Additional	+1	per additional bristle cell group
3°s	Number	+1	per missing 3° cell
Lattice cells	Number	+1	per total lattice cell number above or below twelve (2°s, 3°s and cells of unclear fate are counted; bristles are excluded)

### Cone cell defects

**Figure 2:**
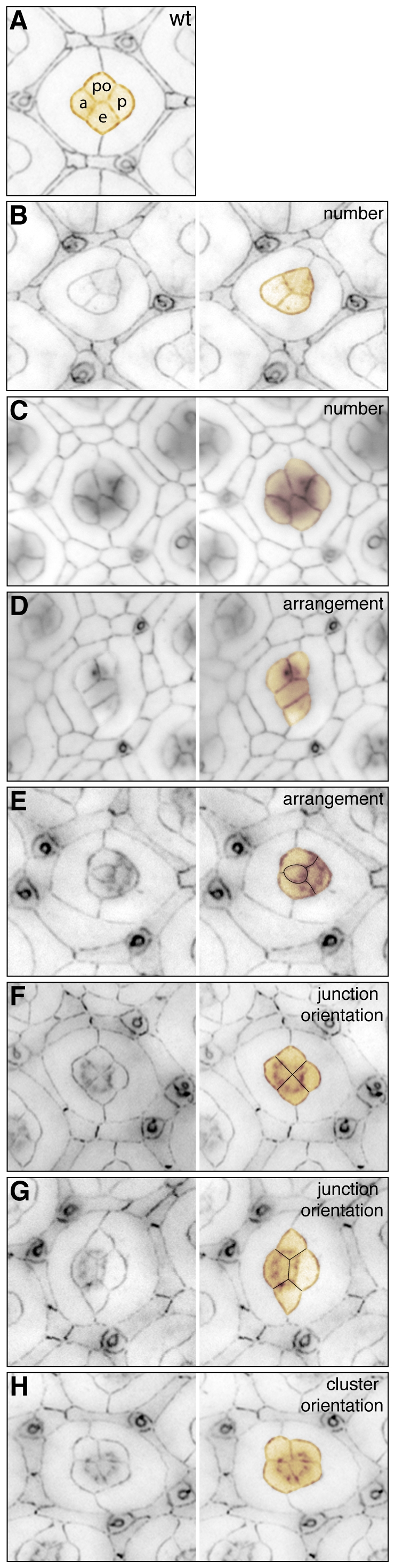
Cone cell errors. A. The final wild type cone cell arrangement, polar (po) and equatorial (e) cells touch, anterior (a) and posterior (p) cells do not. B-H. In mutant tissue defects include loss of cone cells (B), additional cone cells (C), incorrect arrangement of the cells in the cluster (D and E), defects in the orientation of cone cell junctions (F, G), and incorrect orientation of the cluster (H). All tissue was dissected at 41 h APF, cone cells are pseudo-colored orange.

- Four cone cells lie atop wild type ommatidia (Figure 2A). Each field was allocated a point for each missing (Figure 2B) or additional (Figure 2C) cone cell.
- Wild type cone cells arrange in an energetically stable manner that minimizes surface area whilst maximizing adhesion between cone cells [6] (Figure 2A). Any errors in this arrangement (Figure 2D, E) may indicate aberrant junction components or assembly, and were scored by allocating one point.
- As the four cone cells are initially assembled, the anterior and posterior cone cells are in direct contact (Figure 1A). At 18–19 h APF; contact orientation then switches (Figure 1B, C): the polar and equatorial cells contact at the center of the group (Figure 2A). Partial or complete failure to re-orient (Figure 2G and F respectively) was scored by allocating one point.
- Wild type cone cell clusters are precisely oriented within the equatorial-polar plane of the eye epithelium, reflecting proper planar cell polarity. Mis-orientation of the cluster (Figure 2H) was allocated one point.

### Primary pigment cell defects

**Figure 3 pone-0007008-g003:**
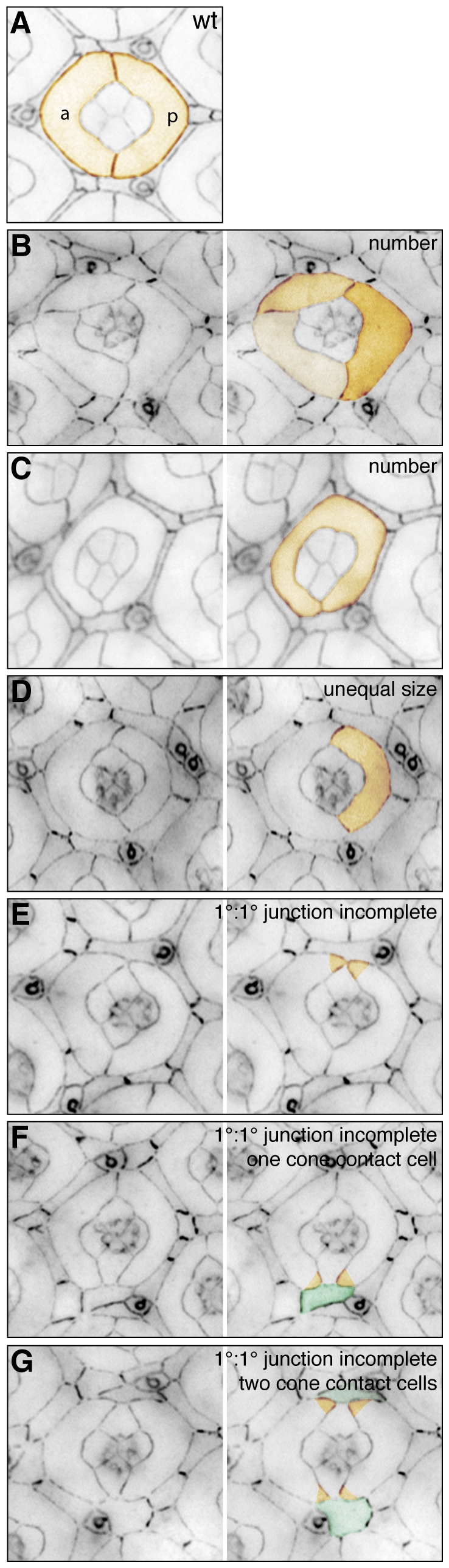
Primary cell errors. A. Anterior (a) and posterior (p) 1° cells make stable junctions enwrapping the central cone cells (pseudo-colored orange). B-G. In mutant tissue additional (B) or too few (C) cells may be recruited to the 1° cell niche, one 1° may be smaller than the other (D), junctions may be incomplete (E-G, only the tips of the two 1°s have been colored for emphasis) allowing lattice cells (colored green in F and G) to directly touch the central cone cell cluster. All tissue was dissected at 41 h APF.

- Two symmetrical 1°s surround the cone cells (Figure 3A). One point was allocated for each additional (Figure 3B) or missing (Figure 3C) 1°. Additional 1°s may indicate ectopic 1° cell recruitment early (16–19 h APF) or later (>19 h APF) due to unstable 1°∶1° cell junctions (see below) or excess 2°/3°s that promote crowding around ommatidia.
- The anterior and posterior 1°s are specified between 16–19 h APF. By 22 h APF, both 1°s are extended around the cone cells and are equal in area. If one 1° was smaller, one point was allocated (Figure 3D). Similarly, if three or more 1°s surrounded the cone cells and were not equal in size one point was allocated.
- Wild type 1°s form stable polar and equatorial boundaries with each other. One point was allocated for any compromise in the fidelity of either of these junctions (Figure 3E-G). To score for the severity of disruption, an additional point was allocated for each interommatidial cell that made direct contact with the central cone cells (termed cone contact cells) consequent to the open 1° phenotype (Figure 3F, G).

### Bristle grouping defects

**Figure 4 pone-0007008-g004:**
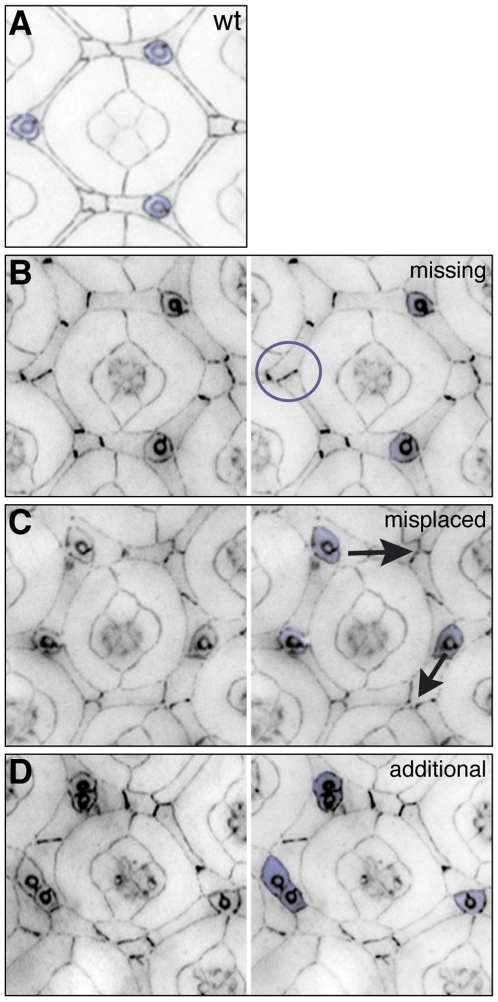
Bristle group errors. A. Three bristle cells (purple) occupy characteristic niches about each ommatidium. B-D. In mutant tissue these may be missing (circles in B), incorrectly placed (arrows in C) or additional bristle groups may be present (D). All tissue was dissected at 41 h APF.

- Bristle organules are comprised of four cells— neuron, glia, trichogen, and tormogen [3]— that are difficult to resolve with standard fluorescence imaging. Hence only gross defects pertaining to overall patterning of the tissue were assessed. Three characteristically positioned bristle groupings surround each wild type ommatidium (Figure 4A); this positioning can be aberrant at the posterior of wild type eye fields but rarely at the center. Points were allocated to fields for each missing (Figure 4B), mis-positioned (Figure 4C) or additional (Figure 4D) bristle group.

### Tertiary pigment cell defects

**Figure 5 pone-0007008-g005:**
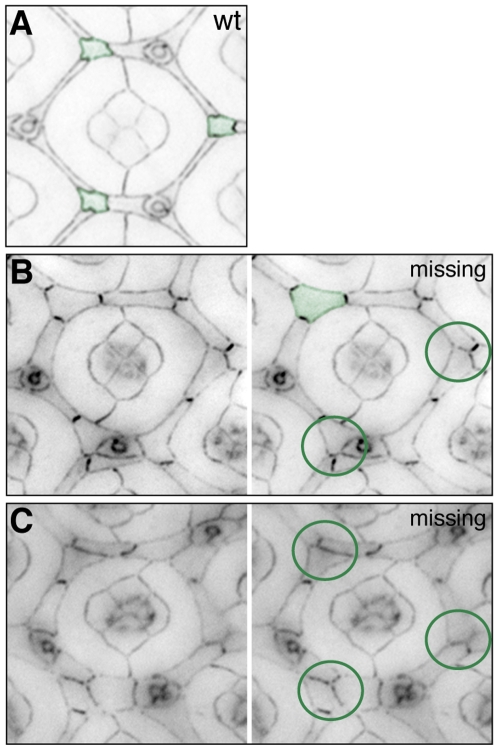
Tertiary cell errors. A. Three 3° cells occupy specific positions in wild type tissue (pseudo-colored green). B and C. In mutant tissue these are frequently not specified (green circles). All tissue was dissected at 41 h APF.

- Individual 3°s inhabit specific niches at the three hexagon vertices surrounding each ommatidium (Figure 5A). A point was allocated per missing 3° cell (Figure 5B, C). A maximum score of three errors for 3° cell defects can be allocated per data point: points were not allocated for additional (more than three) or misplaced 3°'s since these positional defects are accounted for when assessing bristle defects.

### Rotation defects

**Figure 6 pone-0007008-g006:**
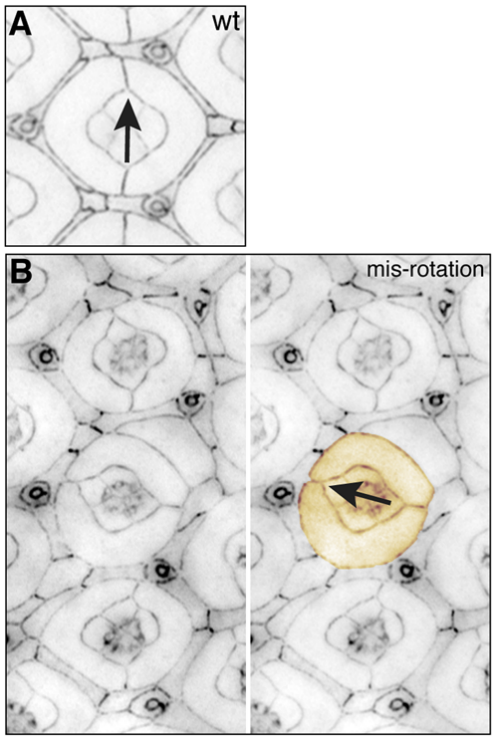
Rotation errors. A. Cone and 1° cells and the underlying ommatidia are precisely oriented along the equatorial-polar axis (arrow). B. In some mutants this orientation is incorrect (orange pseudo-colored group, and arrow), likely the result of incorrect rotation earlier in development. All tissue was dissected at 41 h APF.

- Cone and 1° cells are oriented precisely within the plane of the epithelium (Figure 6A) along an equatorial-polar axis. This arrangement is usually consistent with the arrangement of underlying photoreceptors. Defects in this orientation greater than 30° were termed mis-rotated ommatidia (Figure 6B) and allocated one point. However this classification is arbitrary: whether or not the underlying photoreceptor group and entire ommatidia is mis-rotated is not readily assessed when imaging the apical surface with junctional markers.

### Interommatidial cell number

**Figure 7:**
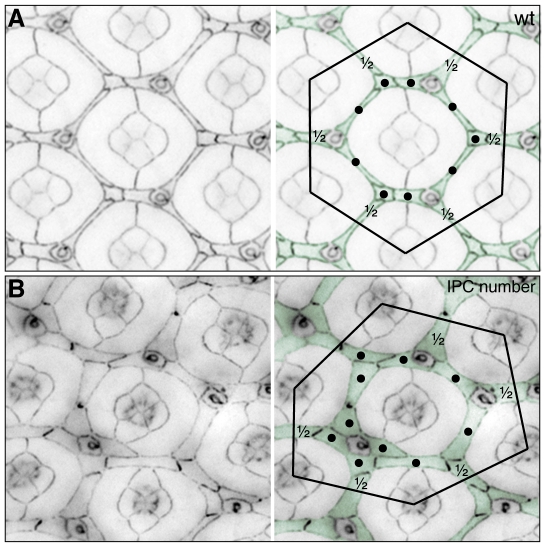
Lattice cell number. A. Twelve cells lie within a data point in wild type tissue: three 2° and 3° cells (labeled with a •) and six half cells (as indicated). B. When a hexagon is superimposed on mutant tissue a change in the number of lattice cells may be observed. All tissue was dissected at 41 h APF; lattice cells are pseudo-colored green.

- In wild type tissue twelve interommatidial pigment cells (IPCs) lie within the hexagonal area of a data point: three 3°s, six complete 2°s, plus the halves of six additional 2°s (illustrated in Figure 7A). For an experimental data point, one point was allocated per additional or missing cell above/below the total of twelve. Cells were counted regardless of their position, size or apparent fate (Figure 7B). Any cells lying partly within the superimposed hexagonal area were scored as half a cell regardless of what proportion of the cell actually lay within the data point area. Additional or missing lattice cells may be consequent to either direct mis-regulation of the apoptotic machinery, signaling to enhance cell survival, or defective cell movements that target cells to specific zones surrounding each ommatidium where apoptosis preferentially occurs [4], [5], [7], [8].
- Patterning of 3°s was additionally scored in our analysis (see above) because this cell fate is dependent on cell signaling, programmed cell death of excess cells, and regulation of adhesion and cytoskeleton. Using live imaging we have observed 3 cells actively compete for this position at each vertex [8]. The 2° cell fate was not separately scored: we consistently find that, provided approximately the correct number of cells are removed by apoptosis and the 3°s are correctly specified, the remaining cells automatically adopt the elongated 2° cell shape. Hence by scoring cell number we also account for the 2° cell fate. In addition, we routinely find that mis-patterning of the ommatidial hexagon is more severe when 3° vertices have not been correctly established than when the 2° niche is mis-specified. We do not directly measure the angles of the hexagonal lattice because distortions are easily introduced during normal tissue processing prior to imaging.

### Example

**Figure 8:**
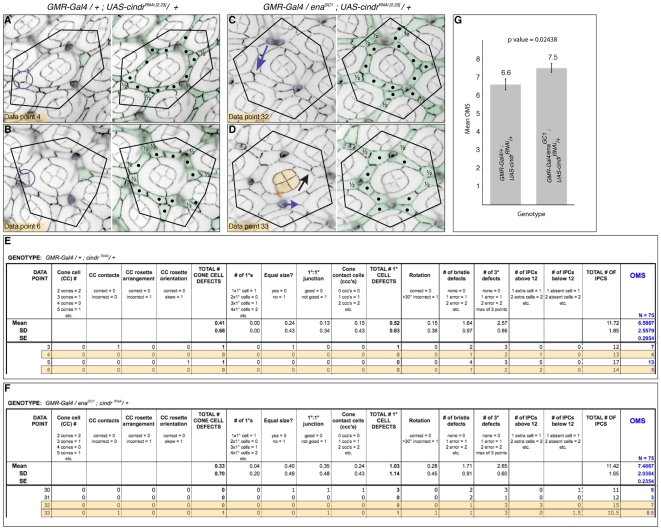
Example: *cindr* interacts with *ena*. A. Data point 4 and B. data point 6 for the *GMR-Gal4/+ ; UAS-cindr^RNA[2.23]^/+* dataset. C. Data point 32 and D. data point 33 for the *GMR-Gal4/ena^GC1^ ; UAS-cindr^RNA[2.23]^/+* dataset. In the left panels cone cells lying within the superimposed hexagons are pseudo-colored purple, circles indicate missing and blue arrows indicate misplaced bristle groups; mis-rotation is indicated by a black arrow; correct 3° cells are indicated in green. All IPCs are colored green in the right panels, whole cells within each hexagonal outline are labeled with a • and half cells indicated. E. and F. Excerpts from our Microsoft Excel databases for each dataset, in each case 4 data points are shown: those provided in panels A to D are highlighted in orange. The scoring system is reiterated in the column headings and Mean and Standard Deviation (SD) values for each feature are shown. Standard error (SE) for the mean OMS is also calculated. G. Graphic comparison of the mean OMS values for each genotype, SE are indicated (bars). The mean OMS values differ significantly at the 5% level (p = 0.02438).

To validate a genetic interaction between the loci for the adaptor protein *cindr* and the actin regulator *enabled* we generated tissue hypomorphic for *cindr* (Fig. 8A and B, *GMR-Gal4/+ ; UAS-cindr^RNA[2.23]^/+*) and in a parallel cross tissue in addition compromised for *ena* dosage (Figure 8C and D, *GMR-Gal4/ena^GC1^; UAS-cindr^RNAi [2.23]^/+*). Images gathered from pupae dissected at 41 h APF were analyzed to generate 75 data points of each genotype. Excerpts from each Excel database are shown in Figure 8E and F.

Specifically, for *GMR-Gal4/+ ; UAS-cindr^RNA [2.23]^*:

Data point 4 (Figure 8A and E):

- no cone or 1° cell defects were observed (though this ommatidium is mildly distorted both 1° cells are equal in size);
- one bristle is missing (blue circle), giving a bristle cell defect score of **1**;
- two 3° cells are missing (the correctly patterned 3° is colored green in the left panel), giving a score of **2** for 3° cell defects.
- a total of thirteen IPCs (colored green in right panel) were counted as opposed to the normal number of twelve, giving a score of **1** (extra cell).
- Hence the total OMS is 1+2+1 = **4**.

Data point 6 (Figure 8B and E):

- one bristle group is missing (blue circle): **1** point;
- two 3° cells are missing: **2** points;
- two additional IPCs are present: **2** points.
- Total OMS = **5**

For *GMR-Gal4/ena^GC1^ ; UAS-cindr^RNA[2.23]^*:

Data point 32 (Figure 8C and F):

- one bristle group is misplaced (blue arrow): **1** point;
- all three 3°s are not specified: **3** points;
- a total of 15 IPCs are present: **3** points.
- Total OMS = **7**

Data point 33 (Figure 8D and F):

- the cone cell contacts are incorrectly oriented: **1** point;
- the lower junction between the 1°sis incomplete (colored orange): **1** point
- the ommatidium is mis-rotated by more than 30°: **1** point
- one bristle group is misplaced: **1** point
- no 3°s have been specified: **3** points
- a total of 10½ IPCs are present (1½ cells missing): **1½** points
- Total OMS = **8½**

The mean OMS values for *GMR-Gal4/+ ; UAS-cindr^RNA[2.23]^/+* and *GMR-Gal4/ena^GC1^; UAS-cindr^RNA[2.23]^/+* were calculated (Figure 8E and F; presented graphically in Figure 8G). A Students T-test was used to compare OMS values of the complete datasets (N = 75) to determine statistical significance: this confirmed that *ena^GC1^* mildly enhanced *cindr^RNAi^* mis-patterning (p-value = 0.02438, significant at the 5% level). In particular the number of rotation and 1° cell defects approximately doubled when *ena* was compromised (compare mean and standard deviations shown in Figure 8E and F) emphasizing that the role of these loci in 1°∶1° junction formation or maintenance, actin regulation and ommatidial rotation warrant further investigation.

## Discussion

Here we present a simple method set to assess apical mis-patterning of the pupal eye. This provides a systemized and unbiased approach useful for quantifying and comparing genotypes. Data sets and derived ommatidial mis-patterning scores are readily assessed for significance using a suitable statistical test if an investigator's aim is to show a clear difference between two or more genotypes (*e.g.*, Student T-test, found in most spreadsheet programs such as Microsoft Excel). Frequently, investigators utilizing the fly pupal eye as an assay have focused on a single aspect of mis-patterning such as interommatidial cell number. However we have found that simply scoring a single component such as cell number does not sufficiently encompass patterning defects and fails to provide a meaningful assessment of mis-patterning. Further, by recording this fuller set of defects, an investigator can additionally evaluate phenotypes specific to one cell type or feature and relate the information to mutants in other loci. Through this approach, we have been able to place loci into functional groups based on the details of their scoring [2] and unpublished data).

## Materials and Methods

### Fly husbandry and genetics

All crosses were cultured as per standard protocols. Pupae were gathered at 0 h APF and cultured at 25°C until dissected as described previously [2].

Genotypes of images presented:

Wild type tissue:

Figure 1, panel A of Figure 2–7: *GMR-Gal4/+ ; UAS-lacZ/+*

Mutant tissue:

Figure 2B and 3C: *GMR-Gal4, UAS-dicer2/+; UAS-klar^RNAi[v32836]^/+* [9]

Figure 2C and 2D: *hsFLP/+; cbl^F165^, FRT80B* [10]

Figure 8C and 8D: *GMR-Gal4/ena^GC1^; UAS-cindr^RNAi [2.23]^/+* [11]

All other panels: *GMR-Gal4/+; UAS-cindr^RNAi [2.23]^/+* [2]

### Imaging

Dissected tissue was fixed and processed as described previously [2]. Rat anti-DE-Cadherin (1∶20, DSHB) was used to visualize adherens junctions. Tissue was imaged using a Leica DM5500 microscope. Images have been minimally processed and pseudo-colored using Photoshop to emphasize specific cell types or features.

### Additional information

Raw images were printed and hexagonal areas superimposed by hand to create data points for scoring as described above. Analyses were recorded in databases generated using Microsoft Excel.
